# A random-permutations-based approach to fast read alignment

**DOI:** 10.1186/1471-2105-14-S5-S8

**Published:** 2013-04-10

**Authors:** Roy Lederman

**Affiliations:** 1 Applied Mathematics Program, Yale University, 51 Prospect st. New Haven, CT 06511, USA

## Abstract

**Background:**

Read alignment is a computational bottleneck in some sequencing projects. Most of the existing software packages for read alignment are based on two algorithmic approaches: prefix-trees and hash-tables. We propose a new approach to read alignment using random permutations of strings.

**Results:**

We present a prototype implementation and experiments performed with simulated and real reads of human DNA. Our experiments indicate that this permutations-based prototype is several times faster than comparable programs for fast read alignment and that it aligns more reads correctly.

**Conclusions:**

This approach may lead to improved speed, sensitivity, and accuracy in read alignment. The algorithm can also be used for specialized alignment applications and it can be extended to other related problems, such as assembly.

More information: http://alignment.commons.yale.edu

## Background

The exponential growth in high-throughput sequencing exceeds the pace of speed increase in computer hardware. Therefore, advancements in software and algorithms for read analysis have been required in order to analyze the tremendous amount of data obtained from sequencing.

Most of the existing programs for read alignment are based on two classes of algorithms: a) prefix-trees (used in programs such as SOAPAligner [1], BWA [2], Bowtie [3] and Bowtie2 [4]) and b) hash-tables (used in programs such as mrFast [5], mrsFast [6], RazerS [7] and Hobbes [8]). Reviews of the main algorithms and software packages developed for read alignment are available in [9-11].

Software packages released in recent years use these approaches very efficiently. When the reference is not very large and not very repetitive, when the number of reads is not large, and when it is possible to "mask" large parts of the reference, existing algorithms and tools provide a computationally inexpensive solution. However, as the throughput continues to grow and new applications emerge, a new approach to read alignment may be useful for many applications.

In this paper, we introduce a random-permutations-based approach to read alignment. The approach is conceptually related to the use of random projections in randomized nearest neighbors algorithms (e.g. [12]). An outline for a random-permutations-based algorithm for string searches has been presented by Charikar [13]. We formulate the read alignment problem as a special nearest neighbors search problem and propose a practical search algorithm based on the random permutations approach.

The applicability of the algorithm is demonstrated by comparing an implementation of the algorithm to existing fast read alignment programs.

### Problem definition

In this subsection we formulate the problem as a "nearest neighbors search" problem.

In the study of the genome, the sequences of nucleotides that form DNA molecules are represented as strings composed of the characters A, C, G and T. We investigate the following scenario: we are given a long reference string (the reference DNA) and a large number of short strings, called "reads." For each of the reads, we would like to find the most similar substring of the reference.

We assume that all our reads are strings of the same length. This assumption often holds in practice, and the approach can be extended to non-uniform lengths. We denote the length of the reads by *M*. A typical value can be *M *≈ 100.

We denote the long reference string, which represents the entire reference genome, by *W*. In the human genome, the length of this string is in the order of *N *≈ 3 × 10^9^.

Instead of considering *W *as one long string, we examine its contiguous substrings of length *M*. There are *N - M *+ 1 such substrings, each of them starts at a different location in *W*. We denote each of these substrings by its location in *W*, so *X_i _*is the substring that begins at the *i*th character of *W *.

We can now phrase our alignment problem as follows: given a read *Y *, find the most "similar" string *X_i _*in the reference library. The measure for similarity is based on the number of mismatches, or the "Hamming distance" between strings; the smaller the distance, the higher the similarity.

This type of search problems (with any definition of distance) is known as "nearest neighbors search."

In this discussion, we describe an algorithm for finding a single, unique, "true nearest neighbor". We assume that no two reference strings are identical. We also assume that there is a unique "true nearest neighbor" for every read, so no other reference string has the same Hamming distance to the read as the "true nearest neighbor." These assumptions simplify the definitions and the analysis, but the approach is applicable when these assumptions do not hold.

### Extended frameworks

The principles discussed in this limited framework and under these assumptions can be extended. For more general search problems, we consider a two-step framework for read alignment: a search step and a refinement step. In the first step, we use a search algorithm to recover candidate alignments and apply a coarse filter to obtain a "small" list of final candidates. In the second step, a more refined method is used to process the list of candidates. This step may include scores (such as the Hamming distance) and thresholds, but may also include cross-referencing of the information recovered from different reads as well as additional searches. This framework is appropriate for permutations-based-algorithms which automatically enumerate many possible candidates.

The prototype presented in the results section implements a version of the algorithm which preforms the first approximate search step and returns a small number of candidates, rather than a single "best" match.

In most of this paper, we restrict our attention to mismatch-type variations and errors. Although considering mismatches is sufficient for some applications, there are other important variations: insertions and deletions ("indels") of characters that change the relative positions of other characters. The implementation in the results section demonstrates one of the extensions for fast and accurate alignment in the presence of indels. A comprehensive discussion of the extensions for indels is beyond the scope of this paper.

### Observations

In the description of the algorithm, we discuss arrays of strings which we sort lexicographically, like words in a dictionary. In particular, we discuss lexicographically sorted libraries containing versions of the strings in the reference library. In this subsection, we describe several properties of lexicographically sorted libraries and properties of strings comparison.

**Definition 1 ***If a string is present in the lexicographically sorted array, we define its "lexicographical position" in the array as its position in the array. If a string is not present in the lexicographically sorted array, we define its "lexicographical position" in the array as the position where it would have been inserted if we had added it to the array*.

**Observation 1 ***There are search algorithms, such as "binary search" *[14], *that allow us to find the lexicographical position of reads in sorted libraries of reference strings in O*(*log*(*N*)) *strings comparison operations. Furthermore, when the reference library contains a perfect match for the read, these search algorithms find the perfect match*.

This operation is very similar to looking up a word in a dictionary.

**Observation 2 ***Suppose that all the mismatches in some read with respect to its true nearest neighbor are known to occur "late enough" in the read, so that the lexicographical position of the read in the sorted array is within K positions from the position of the true nearest neighbor. Then we can find the true nearest neighbor in O*(*log*(*N*) + *K*) *strings comparison operations*.

This can be done by first finding the lexicographical position of the read, and then considering the "neighborhood" of ~ 2*K *strings around it. This operation is analogous to finding the correct page in a dictionary and then examining all the words on that page.

**Observation 3 ***If the same permutation is applied to two strings, the Hamming distance between the permuted strings is equal to the Hamming distance between the original strings*.

A permutation of a string reorders the characters in the string. Therefore, the same mismatches still occur in the permuted versions, only the positions where they occur are changed by the permutations.

## Methods

### An informal description of the algorithm

In our search problem, we have some library of reference strings and a read. Suppose that our read *Y *and its true nearest neighbor *X_i _*have *p *mismatches. Based on observation 3, if we apply the same permutation *π*^(*j*) ^to our read and all the reference strings, the Hamming distance between the permuted version of our read and each permuted reference string is the same as the distance between the original read and the corresponding original reference string. In particular, the number of mismatches between the permuted read *Y*^(*j*) ^and the permuted version of the true nearest neighbor $X_{i}^{\left(j\right)}$ is still *p*, and the permuted version of the true nearest neighbors is the true nearest neighbor of the permuted read. If we are "lucky" enough, we happen to move the *p *mismatches to positions that are "far enough" from the beginning of the string. Based on observation 2, if the positions are "far enough," the search for the lexicographical position of the read leads us to the "neighborhood" of the "true nearest neighbor." Formal definitions of "neighborhoods" are presented below.

We do not know in advance which characters to "push" to the end of the string and we cannot expect to always be "lucky" enough to permute the correct characters away from the beginning. Instead, for each read that we receive, we repeat the procedure described above several times, using a different random permutation each time. Consequently, we have a high probability of finding the true nearest neighbor in at least one of the repetitions.

This procedure uses sorted arrays of permuted strings to define and search for "neighborhoods." Different versions of the algorithm use other data structures, such as prefix-trees of permuted strings.

To illustrate what permutations do, we generated a random "reference genome" of length *N *= 20000, and built a library of all substrings of length 15. In this example, we consider the read *Y *= *CTtGCCAAAGCCATG*, which should be mapped to the location 10000, where *X*_10000 _= *CTCGCCAAAGCCATG*.

We attempt to look for a match to *Y *in the sorted library. Since the mismatch occurred "early" in the read, our search takes us to a distant position in the sorted array and we do not find *X*_10000 _there. The correct neighborhood of *X*_10000 _is presented in table 1.

**Table 1:** Sorted library

Sorted Position	DNA Locus	Original String
9383	8111	CTCGATTGGAACTGA
9384	17930	CTCGCAATCCGCAAA
9385	3710	CTCGCAGTGTCAAAC
9386	1608	CTCGCATCAAAGGTT
9387	10000	CTCGCCAAAGCCATG
9388	17832	CTCGCCCACCTATTA
9389	1034	CTCGCCGGTCTAGTC
9390	19834	CTCGCGCGGTCAACT
9391	6422	CTCGCGTCGGGCGAA

If we permute *Y *using the code *π*^(1)^: (11, 2, 7, 13, 9, 15, 4, 5, 1, 12, 10, 14, 3, 8, 6), the mismatch in position 3 is permuted to the 13th position: *Y*^(1) ^= *π*^(1)^(*Y*) = *CTAAAGGCCCGTtAC*. When we look for *Y*^(1)^, we find the permuted version of *X*_10000_, which is $X_{10000}^{\left(1\right)}$ = *π*^(1) ^(*X*_10000_) = *CTAAAGGCCCGTCAC*. The lexicographical neighborhood of *π*^(1) ^(*X*_10000_) is presented in table 2.

**Table 2:** Sorted library of permuted strings

Sorted Position	DNA Location	Original String	Permuted String
8898	997	ATTACGATAACAACG	CTAAAGACAAACTTG
8899	11316	CTGAGCATAGCTACG	CTAAAGAGCTGCGTC
8900	4844	GTTAGGAAAACAACG	CTAAAGAGGAACTAG
8901	9523	GTGCCCAAATCGATG	CTAAAGCCGGTTGAC
8902	10000	CTCGCCAAAGCCATG	CTAAAGGCCCGTCAC
8903	4568	TTTGTAAGATCTACG	CTAAAGGTTTTCTGA
8904	16699	CTCTCCATAGCCAAG	CTAAAGTCCCGACTC
8905	9139	GTGTCTAGAGCTATG	CTAAAGTCGTGTGGT
8906	1115	GTTTGGAGAGCGAGG	CTAAAGTGGGGGTGG

If we use the same permutation and the mismatch occurs in a different position, we may not find *X*_10000_. In fact, if the mismatch occurs in position 11, it becomes a mismatch in the first character of the permuted string. Therefore, we use several randomly chosen permutations to reduce the probability of failure. When we use long strings and random permutations, the probability of error drops rapidly as the number of iterations grows.

### A more formal description of the algorithm

We now describe the algorithm more formally. First, we describe an indexing procedure (part 1). Then we describe the search for candidate neighbors (part 2). Finally, we describe an approach to filtering the proposed neighbors and finding the best one (part 3).

#### Part 1: Indexes

Create a collection of random permutation schemes {*π*^(*j*)^}.

For each permutation *π*^(*j*)^:

Use *π*^(*j*) ^to permute all the original reference strings.

Build a sorted array *Ar*^(*j*) ^of the permuted reference strings.

Store permutation *π*^(*j*) ^and index *Ar*^(*j*) ^for use in part 2.

End For.

#### Part 2: Lists of candidates

For each read *Y *:

Initialize *Candidates*(*Y *) = ∅.

Randomly choose *J *of the random permutations.

For each chosen permutation:

Calculate *Y*^(*j*) ^= *π*^(*j*)^(*Y *), the permuted version of *Y *.

Find the lexicographical position of *Y*^(*j*) ^in *Ar*^(*j*)^.

Add the lexicographical neighborhood of *Y*^(*j*) ^to *Candidates*(*Y*).

End For.

End For.

#### Part 3: Filter

For each read *Y *:

For every candidate (*i *∈ *Candidates*(*Y *))

Calculate the Hamming distance between *X_i _*and *Y *.

Keep track of the candidate string most similar to the read.

End For.

Report the most similar string as the alignment of *Y *.

End For.

### "Neighborhoods" of strings

In this subsection we give one of the possible definitions for a "neighborhood." We also define the terms "prefix neighborhood" and "resolution length," which we use in the analysis.

We define a neighborhood size *K >*0, which is an order of magnitude of the number of strings that we compare to the read in each iteration.

Suppose that we are looking for the string *Y *in a sorted array of reference strings.

**Definition 2 ***If k is the lexicographical position of the string Y in a sorted array of strings, then the "neighborhood" of Y is defined as the list of strings in positions k *- *K to k *+ *K in the sorted array*.

**Definition 3 ***The "prefix neighborhood of length l" of the string Y is the list of all strings that have the same l-long prefix as the string Y *.

**Definition 4 ***Given a string X, we define the "resolution length" (L) as the smallest value such that the L-long prefix of X is the prefix of no more than K strings in the library*.

### Analysis

In this subsection, we discuss the probability of obtaining the "true nearest neighbor" for a read. We denote the number of mismatches between the read and the true nearest neighbor by *p*.

We assume a constant value of "resolution length" for the true nearest neighbor across the different permutations used. We denote it by *L*. Different reads may have different true nearest neighbors with different values of *L*. This assumption can be relaxed in more detailed analyses of the algorithm. We assume that *p <*(*M - L*).

There are *M*! possible permutations for a string of *M *characters. The permutations that we use are chosen randomly from these *M*! possibilities with equal probabilities.

We begin by considering a single permutation and a single iteration of part 2 of the algorithm. By definition, if there are no mismatches in the first *L *characters of the permuted string, then the true nearest neighbor is in the "neighborhood" and it is added to the list of candidates in this iteration. Since the "neighborhood" examined in part 2 of the algorithm is larger than the "prefix neighborhood of length L," some additional reference strings are added to the list of candidates. There are $\frac{\left(M-L\right)!}{\left(M-L-p\right)!}$ ways to place *p *mismatches in the (*M *- *L*) positions at the end of the string, which are not part of the *L*-long prefix. There are (*M - P*)! ways to place the (*M - P*) characters that have no mismatches. Therefore, there are $\frac{\left(M-L\right)!}{\left(M-L-p\right)!}\left(M-p\right)!$ "lucky" permutations, that permute the *p *mismatches away from the prefix. We assumed that our permutations are chosen from among all *M*! permutations with equal probabilities, so each of the "lucky" permutations is chosen with probability 1/*M*!.

Therefore, the probability of "being lucky" in a single iteration, and adding the true nearest neighbor to the list of candidates is at least:

(1) $$PrGoodPerm\left(p,L,M\right)=\frac{\frac{\left(M-L\right)!}{\left(M-L-p\right)!}\left(M-p\right)!}{M!}.$$

The probability for being "unlucky" in any one experiment is at most 1-PrGoodPerm(p,L,M). The permutations are chosen at random, and they are independent. Therefore, the probability that at least one of the lists contains the true nearest neighbor is:

(2) $$PrSuccess\left(p,L,M,J\right)\geq 1-{\left(1-PrGoodPerm\left(p,L,M\right)\right)}^{J}.$$

In part 3 of the algorithm, we check all the candidates directly, so if the true nearest neighbor is in the list, we are guaranteed to report it as the the best match for the read. So, PrSuccess(p,L,M,J) is also the probability of reporting the correct search result.

Each read has its own true nearest neighbor, therefore the value of *L *and the number of mismatches (*p*) varies between reads. Given a distribution of *L *for the different reads and their "true nearest neighbors," a distribution of the number of mismatches and criteria for the desired probability of success in different cases, we set appropriate values of *K *and *J*. Our experiments suggest that in practice, low values of *K *and *J*, which allow fast computation, can produce good alignment results in a wide range of scenarios.

### Complexity

The indexing of the reference in part 1 of the algorithm is a simple sorting operation which requires *O*(*Nlog*(*N*)) strings comparison operations for each of the indexes.

Based on observations 1 and 2, the number of strings comparison operations required by parts 2 and 3 of the algorithm is *O*(*J*(*log*(*N*) + *K*)) per read.

### Filtration and reporting multiple possible alignments

Since the algorithm evaluates multiple candidates in part 3, some degree of multiple alignments analysis is a byproduct of the algorithm and the algorithm can be extended to report multiple possible alignments. This property allows us to extend the algorithm to perform the fast search required in the first step of the extended alignment framework.

An improved filtration component, the "hit-count" filter, can be used to generate a small list of candidates ("coarse filtration") and also to accelerate the algorithm. A version of the algorithm that uses "hit-counts" stores the number of times each candidate appeared in the searches in part 2 of the algorithm (the "hit-count" for that candidate). In part 3 of this version, the algorithm evaluates and reports only candidates that appeared in several searches in different indexes ("received multiple hits").

### Memory considerations and practical indexes

Large reference genomes may require multiple large indexes. It is enough to store the original reference string and the permutation rules, and it is not necessary to store all the permuted strings explicitly in the sorted arrays. It is also not necessary to store all the indexes in the RAM at the same time; one can load an index, perform one iteration of part 2 of the algorithm for a batch of reads, and then load another index.

Furthermore, each single index can be used *almost *as if it were multiple indexes with different permutations. To achieve this, we use a sliding window to take contiguous substrings of the reads. We permute each of these substrings and search for it in the sorted array of permuted reference strings.

## Results and discussion

### Basic alignment

We implemented a version of the algorithm in C (with no SIMD/SSE). Our permutations-based prototype implementation was used in the same three modes in all the experiments.

For the comparison presented here, we chose some of the popular programs which preform the fastest alignment to a human reference genome. We used Bowtie [3] as the main benchmark for performance evaluation because it is one of the fastest aligners [11]. We also compared the performance to BWA [2] and the more recent Bowtie2 [4].

The purpose of the comparison is to demonstrate the applicability of the algorithm to a large-scale problem like aligning to a full human genome. This is not meant to be a complete comparison to all programs in all scenarios.

All the real reads were obtained from The 1000 Genomes Project [15]. All the simulated reads were produced using wgsim [16]. The human genome GRCh37 [17] (obtained from The 1000 Genomes Project) was used as the reference. Some large regions were masked with "N"s in the original reference, but other repetitive regions were not masked.

The comparison was performed on a cluster node with (2) E5620 CPUs and 48 GB RAM. Similar experiments of alignment to the full human genome, using low-cost ($500-600) desktops with 16 GB and 32 GB RAM, produced similar results. All the programs were used in single thread mode. Bowtie requires about 2.2 GB of RAM, Bowtie2 requires about 3.1 GB and BWA requires about 2.3 GB. The permutations-based prototype implementation requires about 15 GB of RAM for the full human genome (Using more memory would allow to index the reverse complement, doubling the speed. There is a 8 GB version of the program for computers with smaller RAM. Smaller references require smaller indexes).

**Figure 1:**
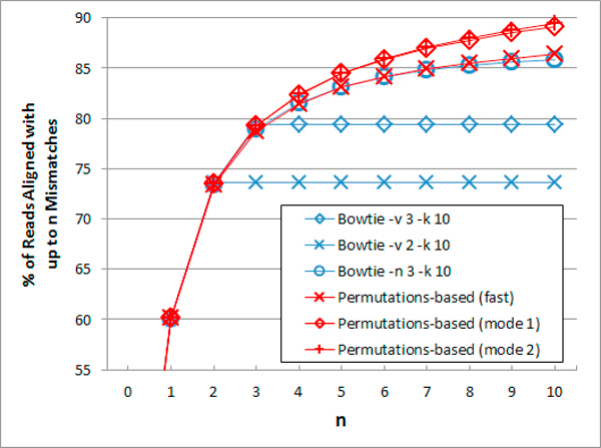
**Single-end alignment of real reads: best match**. The percent of reads for which an alignment with up to n mismatches was found. Additional alignments are ignored. Dataset: 105 reads from ERR009392_1.

In Figure 1 we compare the best alignments obtained by Bowtie and the permutations-based prototype. In certain settings, Bowtie found alignments with fewer mismatches for some reads. For example, "Bowtie -v 3" found alignments with up to 3 mismatches for about 0.1% more reads (not visible in the figure). The permutations-based prototype found more alignments for reads with a large number of mismatches than all the modes which we tested in this experiment (most modes are not shown in the figure), and found more alignments with a low number of mismatches than some modes of Bowtie (the default "-n" modes).

In table 3 we compare the search times of Bowtie, Bowtie2, BWA and the permutations-based prototype in alignment of real reads. The different programs have different criteria for reporting, and the permutations-based prototype usually generates more possible alignments, so the number of alignments would be misleading and it is not reported in the tables.

**Table 3:** Real single-end reads: search time

Software	Search time (s)		
	**SRR023337_1** **78 bp**	**ERR009392_1** **108 bp**	**ERR016249_1** **160 bp**

Bowtie -v 3	233	256	773

Bowtie -n 2	144	334	1560

Bowtie -n 2 -k 10	658	1142	2830

Bowtie2 -very-fast	179	285	440

Bowtie2 -sensitive	328	654	853

Bowtie2 -very-sensitive	812	1488	1855

Bowtie2 -very-sensitive -k 10	1121	2430	3869

BWA -o 0	548	860	2434

Permutations-based (mode 1)	65	68	111

Permutations-based (mode 2)	147	151	145

Permutations-based (fast)	**35**	**39**	**57**

Each dataset contained 10^6 ^reads from the fastq files obtained from the "1000 Genomes" project.

**Figure 2:**
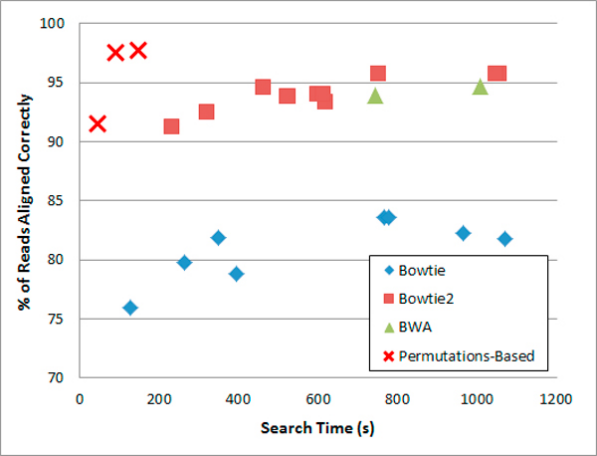
**Single-end alignment of simulated reads: search time and correct alignments**. Dataset: 106 simulated reads of length 100, mutation rate: 0:1%, indel ratio: 15%, mismatch rate: 2%. The results of additional simulations are reported in table 4.

We also simulated reads to measure how many of the reads are aligned to the "correct original location in the genome." In some cases, there may be several equally probable alignments and in some cases the "best" alignment with the fewest mismatches may be different than the "correct location." Ideally, the alignment program should report both the "best" alignment and other possibilities that could be the "correct" alignment. The results of this experiment are presented in Figure 2 and table 4. In most cases, the permutations-based prototype was faster than Bowtie, Bowtie2 and BWA and produced more correct alignments to the correct "original location" than all other programs.

**Table 4:** Simulated single-end reads: search time and percent of correct alignments

Read length		75			100			150		
**Mismatch probability**		**1%**	**2%**	**5%**	**1%**	**2%**	**5%**	**1%**	**2%**	**5%**

Software	Time/%Correct									

Bowtie -v 3	time (s) % correct	141 94.9	245 89.0	517 45.0	177 94.4	350 81.9	588 24.1	306 89.5	537 60.9	647 5.0

Bowtie -v 2	time (s) % correct	58 91.1	90 76.2	165 25.1	79 87.6	125 63.7	190 11.0	126 76.2	194 38.9	218 1.6

Bowtie -n 3	time (s) % correct	152 92.6	236 84.6	439 67.7	234 92.5	393 78.8	961 65.2	397 88.2	733 59.4	2430 41.9

Bowtie -n 2	time (s) % correct	108 93.1	161 85.7	286 69.6	170 93.0	264 79.7	609 67.0	294 88.6	514 59.9	1560 43.1

Bowtie -n 2 -k 10	time (s) % correct	873 96.2	869 88.7	1179 72.9	1242 95.3	1069 81.7	2377 69.2	1511 90.3	1243 61.0	3446 44.0

Bowtie2 -very-fast	time (s) % correct	169 93.6	163 87.3	136 59.5	234 95.3	233 91.2	196 69.4	363 95.9	366 93.0	299 76.1

Bowtie2 -fast	time (s) % correct	224 94.0	223 88.2	178 60.8	329 95.9	320 92.4	267 71.0	533 96.9	523 94.7	422 78.2

Bowtie2 -sensitive	time (s) % correct	354 95.2	315 92.1	275 73.1	498 96.5	462 94.6	396 80.4	791 97.6	765 96.6	638 88.4

Bowtie2 -very sensitive	time (s) % correct	776 95.7	734 94.2	611 83.6	1124 96.8	1056 95.8	851 89.1	1864 97.8	1730 97.3	1383 94.2

Bowtie2 -very sensitive -k 10	time (s) % correct	1063 **98.0**	813 95.8	691 84.6	1743 98.4	1341 96.6	1165 89.9	3498 98.9	2775 97.6	2395 **94.9**

BWA -o 0	time (s) % correct	320 96.5	457 93.3	516 59.9	404 97.2	743 93.9	610 54.2	715 97.4	931 92.5	447 33.1

BWA -o 1	time (s) % correct	359 97.1	560 93.8	889 60.2	483 98.1	1007 94.7	1206 54.6	904 **98.7**	1513 93.6	1034 33.4

Permutations-based (mode 1)	time (s) % correct	53 97.5	76 95.8	106 84.5	56 98.4	90 97.5	118 89.0	76 98.5	98 98.0	112 91.3

Permutations-based (mode 2)	time (s) % correct	147 97.8	149 **96.4**	152 **86.6**	155 **98.5**	147 **97.8**	145 **90.6**	145 98.6	148 **98.1**	156 92.7

Permutations-based (fast)	time (s) % correct	**31** 93.7	**43** 88.5	**62** 64.1	**33** 95.8	**46** 91.5	**64** 66.9	**45** 96.9	**51** 93.3	**72** 69.4

Each dataset contained 10^6 ^reads. Mutation rate: 0.1%, indel ratio: 15%.

We report the search time (for BWA: overall run time) and the percent of the reads which were aligned to the correct position in the genome.

In some cases and in some settings, the programs may report several possible alignments for some reads. When needed, additional filtering can be added to aligners in order to eliminate some of the results, as appropriate for specific applications. In the experiment, the programs reported *<*4 alignments/read (in average, in sensitive modes), with 0-1 alignments for the majority of reads. In this table, when the program produces multiple possible alignments, it is enough that one of the reported alignments corresponds to the correct location in order to consider the alignment correct.

### Alignment of paired-end reads, in the presence of indels

One common variation in the alignment scenario we described is the use of a different type of distance: "edit distance." Strings may be close in "edit distance" even in the presence of indels, although the indels are likely to make the strings far apart in Hamming distance.

Another common variation in the scenario is the availability of "paired-end reads": reads are presented in pairs, in which both reads are known to have originated from nearby areas in the genome. In this case, we are required to find nearest neighbors for both strings, subject to some constraint on the distance between the locations in the reference genome.

A modified version of our prototype performs paired-end alignment in the presence of indels. We first align each of the reads in the pair separately. Then, for each "reasonable" candidate found for one of the reads, we restrict our attention to the area of the reference genome around that candidate, and attempt to align substrings of the other read to that area.

Since Bowtie does not allow indels, we used Bowtie2 [4] and BWA [2] as benchmarks. All the programs were used in single thread mode. This version of the prototype requires about 25 GB of RAM for the alignment of paired-end reads to a human genome. Bowtie requires about 2.9 GB of RAM, Bowtie2 requires about 3.2 GB and BWA requires about 2.3 GB.

In table 5 we compare the search times of Bowtie, Bowtie2, BWA and the permutations-based prototype implementation in paired-end alignment of real reads.

**Table 5:** Real paired-end reads: search times.

Software	Search time (s)	
	**SRR023337** **78 bp, paired**	**ERR009392** **108 bp, paired**

Bowtie -v 3	2004	2145

Bowtie -v 2	**264**	315

Bowtie -n 3	628	718

Bowtie2 -very-fast	650	848

Bowtie2 -fast	740	961

Bowtie2 -sensitive	978	1351

Bowtie2 -very-sensitive	1749	2576

BWA -o 0	903	2001

BWA -o 1	1707	3540

Permutations-based (report one)	345	**259**

Permutations-based (report more)	608	488

Each dataset contained 10^6 ^pairs of reads from the fastq files obtained from the "1000 Genomes" website. Search times are reported.

**Figure 3:**
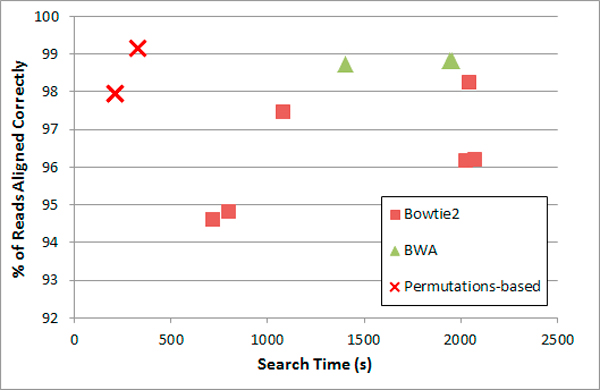
**Paired-end alignment of simulated reads: search time and correct alignments**. Dataset: 106 pairs of reads of length 100, mutation rate: 0:1%, indel ratio: 15%, mismatch rate: 2%. Additional results are reported in table 6.

In Figure 3 and table 6 we present the results of aligning simulated pairs of reads with indels. The permutations-based prototype was faster than the other programs and usually produced more correct alignments when there were few indels. The permutations-based prototype was also able to align reads with higher indel rates almost as well as the best performing benchmark program, but significantly faster.

**Table 6:** Simulated paired-end reads: search time and percent of correct alignments.

		Low indel probability			High indel probability		
**Mismatch probability**		**1%**	**2%**	**5%**	**1%**	**2%**	**5%**

Software	Time/%Correct						

Bowtie -v 3	time (s) %correct	1766 91.8	2274 69.3	3068 6.1	1849 83.2	2375 63.8	3059 5.7

Bowtie -v 3 -k 10	time (s) %correct	6161 93.6	5283 70.5	3260 6.1	5793 84.9	5151 64.9	3246 5.8

Bowtie -v 2	time (s) %correct	241 79.7	333 42.2	353 1.3	256 73.2	337 39.6	352 1.2

Bowtie -n 2	time (s) %correct	483 86.4	593 64.8	645 50.4	486 78.4	618 59.6	664 46.1

Bowtie -n 2 -k 10	time (s) %correct	1308 87.8	1293 65.7	1054 51.1	1283 79.6	1199 60.5	1036 46.8

Bowtie2 -very-fast	time (s) %correct	757 98	715 94.6	506 67.0	734 98.0	670 94.6	484 67.0

Bowtie2 -fast	time (s) %correct	834 98.1	800 94.8	571 67.5	804 98.1	750 94.8	551 67.5

Bowtie2 -sensitive	time (s) %correct	1104 98.4	1079 97.5	893 83.8	1062 98.4	1014 97.5	857 83.8

Bowtie2 -very-sensitive	time (s) %correct	2070 98.5	2045 98.3	1671 **96.5**	2080 98.5	1968 98.3	1648 **96.5**

BWA -o 0	time (s) %correct	1026 99.1	1404 98.7	1670 78.1	1162 98.6	1379 98.0	1676 76.3

BWA -o 1	time (s) %correct	1252 99.1	1956 98.8	3062 78.4	1499 **99.1**	2274 **98.8**	3180 78.0

Permutations-based (report one)	time (s) %correct	**155** 98.2	**208** 97.9	**300** 95.0	**166** 97.6	**223** 97.2	**313** 93.6

Permutations-based (report more)	time (s) %correct	227 **99.5**	328 **99.2**	530 **96.5**	268 98.9	365 98.5	553 95.2

The "low indel probability" datasets were generated with mutation rate: 0.1%, and indel ratio: 15%. The "high indel probability" datasets were generated with mutation rate: 0.1% and indel ratio: 100%. Each of the datasets contains 10^6 ^pairs of 100 character-long reads.

For each dataset and each program, we report the search time (for BWA: overall run time) and the percent of the reads which were aligned to the correct position in the genome.

In some cases and in some settings, the programs may report several possible alignments for some reads. When needed, additional filtering can be added to aligners in order to eliminate some of the results, as appropriate for specific applications. In the experiment, the programs reported *<*3 alignments/read (in average, in sensitive modes), with 0-1 alignments for the majority of reads. In this table, when the program produces multiple possible alignments, it is enough that one of the reported alignments corresponds to the correct location in order to consider the alignment correct.

## Conclusions

An algorithm has been constructed for the fast alignment of DNA reads to a reference genome. The algorithm handles mismatches by design, and it has been demonstrated that it can be extended to allow some inserts and deletions in some cases of practical interest.

The algorithm has been implemented and compared to existing programs. Our experiments indicate that the algorithm can produce alignments comparable to those generated by existing fast alignment algorithms, often aligning more reads with a significant speed increase. Future implementations of the algorithm are expected to be faster and more efficient, sensitive and accurate.

The permutations-based prototype implementation requires 15 GB of RAM for the alignment of reads to a human genome (25 GB for paired-end alignment). Some existing programs require significantly less memory. However, the amount of memory required by this implementation is available in low cost computers, and other versions of the algorithm utilize memory more efficiently.

Our current implementation of the algorithm is not a complete software package and does not replace existing software packages. This prototype implementation demonstrates how the proposed algorithm can be used to enhance existing software packages and to build new software packages.

The scope of this discussion is limited to the basic problem of fast alignment to large genomes. Separate work on this class of algorithms indicates that the algorithms can also be used for very fast alignment of long 454 and Ion Torrent reads which may have many indels. Other work indicates that these algorithms can be used for other applications, such as assembly. Additional preliminary results and technical reports are available at http://alignment.commons.yale.edu.

## Competing interests

The author developed patent-pending methods for processing in sequencing.

## Declarations

Publication of this article was supported by the author.

This article has been published as part of *BMC Bioinformatics *Volume 14 Supplement 5, 2013: Proceedings of the Third Annual RECOMB Satellite Workshop on Massively Parallel Sequencing (RECOMB-seq 2013). The full contents of the supplement are available online at http://www.biomedcentral.com/bmcbioinformatics/supplements/14/S5.

## References

[B1] Li R Yu C Li Y Lam T Yiu S Kristiansen K Wang J SOAP2: an improved ultrafast tool for short read alignment Bioinformatics 2009 25 15 1966 1967 10.1093/bioinformatics/btp336 19497933

[B2] Li H Durbin R Fast and accurate short read alignment with Burrows-Wheeler transform Bioinformatics 2009 25 14 1754 1760 http://bioinformatics.oxfordjournals.org/content/25/14/1754 10.1093/bioinformatics/btp324 19451168 PMC2705234

[B3] Langmead B Trapnell C Pop M Salzberg S Ultrafast and memory-efficient alignment of short DNA sequences to the human genome Genome Biol 2009 10 3 R25 10.1186/gb-2009-10-3-r25 19261174 PMC2690996

[B4] Langmead B Salzberg S Fast gapped-read alignment with Bowtie 2 Nature methods 2012 9 4 357 359 10.1038/nmeth.1923 22388286 PMC3322381

[B5] Alkan C Kidd JM Marques-Bonet T Aksay G Antonacci F Hormozdiari F Kitzman JO Baker C Malig M Mutlu O Sahinalp SC Gibbs RA Eichler EE Personalized copy number and segmental duplication maps using next-generation sequencing Nature Genetics 2009 41 10 1061 1067 http://www.nature.com/doifinder/10.1038/ng.437 10.1038/ng.437 19718026 PMC2875196

[B6] Hach F Hormozdiari F Alkan C Hormozdiari F Birol I Eichler E Sahinalp S mrsFAST: a cache-oblivious algorithm for short-read mapping Nature methods 2010 7 8 576 577 10.1038/nmeth0810-576 20676076 PMC3115707

[B7] Weese D Emde A Rausch T Döring A Reinert K RazerS--fast read mapping with sensitivity control Genome Research 2009 19 9 1646 1654 10.1101/gr.088823.108 19592482 PMC2752123

[B8] Ahmadi A Behm A Honnalli N Li C Weng L Xie X Hobbes: optimized gram-based methods for efficient read alignment Nucleic Acids Research 2012 40 6 e41 e41 http://nar.oxfordjournals.org/content/40/6/e41.abstract 10.1093/nar/gkr1246 22199254 PMC3315303

[B9] Li H Homer N A survey of sequence alignment algorithms for next-generation sequencing Briefings in Bioinformatics 2010 11 5 473 483 10.1093/bib/bbq015 20460430 PMC2943993

[B10] Flicek P Birney E Sense from sequence reads: methods for alignment and assembly Nature Methods 2009 6 S6 S12 10.1038/nmeth.1376 19844229

[B11] Fonseca NA Rung J Brazma A Marioni JC Tools for mapping high-throughput sequencing data Bioinformatics 2012 28 24 3169 3177 http://bioinformatics.oxfordjournals.org/content/28/24/3169 10.1093/bioinformatics/bts605 23060614

[B12] Jones P Osipov A Rokhlin V A randomized approximate nearest neighbors algorithm Applied and Computational Harmonic Analysis 2012 21885738 10.1073/pnas.1107769108 PMC3179075

[B13] Charikar M Similarity estimation techniques from rounding algorithms Applied and Computational Harmonic Analysis 2002 ACM 380 388

[B14] Black PE Dictionary of Algorithms and Data Structures 2012 U.S. National Institute of Standards and Technology http://xlinux.nist.gov/dads/

[B15] Altshuler D Lander E Ambrogio L Bloom T Cibulskis K Fennell T Gabriel S Jaffe D Sheer E Sougnez C A map of human genome variation from population scale sequencing Nature 2010 467 7319 1061 1073 10.1038/nature09534 20981092 PMC3042601

[B16] Li H Wgsim https://github.com/lh3/wgsim

[B17] Collins F Lander E Rogers J Waterston R Conso I Finishing the euchromatic sequence of the human genome Nature 2004 431 7011 931 945 10.1038/nature03001 15496913

